# Taxifolin, an Inhibitor of Sortase A, Interferes With the Adhesion of Methicillin-Resistant *Staphylococcal aureus*

**DOI:** 10.3389/fmicb.2021.686864

**Published:** 2021-07-06

**Authors:** Li Wang, Guangming Wang, Han Qu, Kai Wang, Shisong Jing, Shuhan Guan, Liyan Su, Qianxue Li, Dacheng Wang

**Affiliations:** ^1^College of Animal Science, Jilin University, Changchun, China; ^2^Department of Neurosurgery, The First Hospital of Jilin University, Changchun, China; ^3^Changchun Veterinary Research Institute, Chinese Academy of Agricultural Sciences, Changchun, China; ^4^College of Veterinary Medicine, Jilin University, Changchun, China

**Keywords:** antivirulence, taxifolin, inhibitor, methicillin-resistant *Staphylococcus aureus*, sortase A, pneumonia

## Abstract

The evolution and spread of methicillin-resistant *Staphylococcus aureus* (MRSA) poses a significant hidden risk to human public health. The majority of antibiotics used clinically have become mostly ineffective, and so the development of novel anti-infection strategies is urgently required. Since *Staphylococcus aureus* (*S. aureus*) cysteine transpeptidase sortase A (SrtA) mediates the surface-anchoring of proteins to its surface, compounds that inhibit SrtA are considered potential antivirulence treatments. Herein, we report on the efficacy of the potent SrtA inhibitor taxifolin (Tax), a flavonoid compound isolated from Chinese herbs. It was able to reversibly block the activity of SrtA with an IC_50_ of 24.53 ± 0.42 μM. Tax did not display toxicity toward mammalian cells or *S. aureus* at a concentration of 200 μM. In addition, Tax attenuated the virulence-related phenotype of SrtA *in vitro* by decreasing the adherence of *S. aureus*, reducing the formation of a biofilm, and anchoring of *S. aureus* protein A on its cell wall. The mechanism of the SrtA-Tax interaction was determined using a localized surface plasmon resonance assay. Subsequent mechanistic studies confirmed that Asp-170 and Gln-172 were the principal sites on SrtA with which it binds to Tax. Importantly, *in vivo* experiments demonstrated that Tax protects mice against pneumonia induced by lethal doses of MRSA, significantly improving their survival rate and reducing the number of viable *S. aureus* in the lung tissue. The present study indicates that Tax is a useful pioneer compound for the development of novel agents against *S. aureus* infections.

## Introduction

There is currently major worldwide concern about the continuous emergence of multidrug-resistant bacterial pathogens. *Staphylococcus aureus* (*S. aureus*) is recognized as an important cause of disease worldwide. It is capable of inducing a variety of serious diseases that endanger human health, from mild skin and soft tissue infection to fatal invasive infections such as septicemia and pneumonia, etc. (Goetghebeur et al., 2007; Tong et al., 2015; Lake et al., 2018). In clinics, antibiotics have been considered as the primary defense against *S. aureus* infections over many decades. In the years following their introduction, the therapeutic benefits of such antibiotics were remarkable. However, the considerable selective pressure and improper use of antibiotics have resulted in the emergence, prevalence, and spread of drug-resistant strains of bacteria (Liu et al., 2019; Wu et al., 2019). As strains of methicillin-resistant *Staphylococcus aureus* (MRSA) with little sensitivity to conventional antibiotics have become prevalent (Prestinaci et al., 2015), treatments for MRSA infection have become more challenging for clinicians, who require new strategies to provide effective therapeutic options against complicated *S. aureus* infections (Kali, 2015; Galar et al., 2019).

Alternative therapies such as the use of antibiotics in combination or with adjuvants, bacteriophages, antimicrobial peptides, nanoparticles (Nisar et al., 2019) and anti-virulence therapy are widely reported (Mandal et al., 2014; Kaur, 2016). Considering that *S. aureus* exploited a vast repertoire of virulence strategies that enable it to infect a host. Therefore, Therefore, exploration of the potential of these virulence factors as drug targets may represent an alternative approach to disrupt bacterial pathogenicity (Vandenesch et al., 2012). SrtA is a membrane-bound cysteine transpeptidase that plays an essential role in catalyzing the covalent anchoring of surface proteins to the bacterial cell wall (Paterson and Mitchell, 2004). A member of the sortase subfamily, it plays an active role in bacterial adhesion, biofilm formation, and immune escape (Cascioferro et al., 2014). In addition, *S. aureus* SrtA-anchored surface proteins play major roles in the infection process, with many studies demonstrating that *S. aureus* SrtA mutants do not form abscess lesions or survive when infecting mouse tissue (Mazmanian et al., 2000; Cheng et al., 2009). Specific SrtA inhibitors do not interfere with the growth of bacteria yet weaken bacterial virulence (Suree et al., 2007; Hou et al., 2018). They have the potential to prevent *S. aureus-*induced colonization and invasive diseases while exhibiting a low risk of causing bacterial resistance by blocking SrtA (Hou et al., 2018).

Novel SrtA inhibitors have been identified from libraries of natural or synthetic compounds, or may be specifically-engineered peptidomimetics (Smeltzer, 2016). Of these, there is widespread interest in natural products with diverse structures and biological properties from fungi and plants (Si et al., 2016). In the present study, characterization of the natural compound taxifolin (Tax) is reported. It is extracted from the roots of larch and is an inhibitor of SrtA. It has been widely studied due to its properties as an antioxidant and inhibitor of the synthesis of fat and cholesterol (Angelis et al., 2016; Razak et al., 2018; Ren et al., 2020). In addition, it has been observed that Tax can inhibit SrtA activity without interfering with the growth of bacteria, suggesting that the use of Tax would result in low selective pressure which would avoid the development of resistant strains. In the present study, the inhibitory mechanisms were investigated in detail and the protective effects of Tax in a murine model of MRSA-induced lethal pneumonia was evaluated. In conclusion, the results indicated that Tax represents a potential anti-MRSA drug.

## Materials and Methods

### Reagents and Materials

The peptide substrate Abz-LPATG-Dap (Dnp)-NH_2_ (Abz:ortho-aminobenzoic acid; Dnp:2,4-dinitrophenyl) was provided by LifeTein (Beijing, China). Dimethyl sulfoxide (DMSO) was purchased from Beyotime (Shanghai, China). A library containing 420 natural compounds derived from Chinese traditional herbs was purchased from Pufeide Biotech Company (Chengdu, China).

### Bacteria and Growth Conditions

*S. aureus* USA300 was obtained from the American Type Culture Collection (Manassas, VA). The *S. aureus* Newman SrtA deletion mutant (Δ*srtA*) and the pET28a-SrtA strain were already used and stored within the laboratory. *E. coli* BL21 (DE3) was used as a host for protein expression and purchased from the TransGen Biotech (Beijing, China). *E. coli* and *S. aureus* were cultured in Luria-Bertani broth (LB, Hopebio, Qingdao, China) and brain heart infusion medium (BHI, Solarbio, Beijing, China), respectively, at 37°C with constant shaking.

### Expression and Purification of Recombinant SrtA and Its Mutants

Site-directed mutagenesis for D170A-SrtA and Q172A-SrtA was performed based on plasmid pET28a-SrtA using a multi-site mutagenesis kit (Transgen, Beijing, China) and the desired mutation was verified via DNA sequencing by Sangon Biotech (Shanghai, China). All primers used in the study are presented in Table 1. Subsequently, recombinant SrtA and the SrtA mutant proteins (D170A and Q172A) were expressed and purified in accordance with a previously published procedure (Zhulenkovs et al., 2014). Briefly, bacteria were cultured until an OD_600_ value of 0.8 was achieved, after which 0.5 mM isopropyl-β-D-thiogalactopyranoside (IPTG) was added to induce recombination of SrtA at 16°C overnight. Because the recombinant protein had 6 × His tags, it was purified using a nickel-nitrilotriacetic acid (Ni-NTA) purification system. Imidazole (10 mM) was used to wash away excess protein, while 400 mM imidazole was employed to elute the target protein.

**TABLE 1:** Primers used in this study.

**Primer name**	**Sequences (5′–3′)**
D170A -*srtA*-F	GAGTTCTAGCTGAACAAAAAGG
D170A -*srtA*-R	CTACATCTGTAGGCTTAACATC
Q172A -*srtA*-F	CTAGATGAAGCAAAAGGTAAAG
Q172A -*srtA*-R	AACTCCTACATCTGTAGGCTTA

### Screening of SrtA Inhibitors

Fluorescence resonant energy transfer (FRET) was used to determine the effect of Tax on SrtA, as described previously (Kruger et al., 2002). Briefly, 4 μM purified recombinant SrtA was combined with various concentrations of Tax then added to assay buffer consisting of 50 mM Tris-HCl, 5 mM CaCl_2_, 150 mM NaCl, at pH 7.5, to a final volume of 200 μL. Following incubation of the mixture at 37°C for 1 h, substrate peptide was added to a final concentration of 10 μM and incubated for an additional 30 min. Fluorescence intensity was measured at excitation and emission wavelengths of 309 and 420 nm, respectively.

### Susceptibility Assay and Growth Curve

A broth microdilution assay was performed to determine the minimum inhibitory concentration (MIC) of Tax for *S. aureus* USA300, as described elsewhere (Sader et al., 2013; Delgado-Valverde et al., 2017). A growth curve was further evaluated by adding various concentrations of Tax (0–200 μM) to a bacterial culture, then incubating until an OD_600_ of 0.3 had been reached. *S. aureus* USA300 and Δ*srtA* were used as controls. The OD_600_ value was recorded for each sample at 1 h intervals for a total of 24 h. The growth rate between *S. aureus* treated with Tax and *S. aureus* USA300 was also calculated at each time point.

### Eukaryotic Cytotoxicity

Cytotoxicity was determined using a cell counting kit-8 assay (CCK-8, US EVERBRIGHT, Suzhou, China), as described previously (Xiao et al., 2018). HEK293 or HepG2 cells were seeded in 96-well plates (Corning, United States) at a density of 5 × 10^4^ cells/well, then incubated at 37°C in an atmosphere containing 5% CO_2_ for 24 h. The culture medium was then exchanged with fresh medium containing different concentrations of Tax (0–200 μM) or vehicle and incubated for an additional 24 h. CCK-8 solution (10 μL) was carefully added then incubated for 4 h in an incubator at 37°C. The OD value at 450 nm was measured to calculate cell viability.

### Fibrinogen Binding Assay

Bacterial cultures grown overnight were diluted (1:100) in fresh BHI medium containing different concentrations of Tax then cultured at 37°C until an OD_600_ of 1.0 had been achieved. The Δ*srtA* strain was used as a positive control. Aliquots of bacteria were placed in the wells of a 96-well plate pre-coated with bovine fibrinogen (20 μg/mL). After incubation at 37°C for 2 h, the cell suspension in each well was replaced with 100 μL of 25% formaldehyde then incubated for a further 30 min, after which the formaldehyde was discarded. The plates were washed twice with PBS, and then 0.1% (w/v) crystal violet was added to stain the cells. Finally, after 20 min, the wells were gently washed with PBS then dried, and the OD value at 570 nm was measured.

### Effects of Tax on Biofilm Formation

Bacterial cultures grown overnight were diluted 1:100 in BHI medium supplemented with 3% NaCl and 0.5% glucose. A 200 μL aliquot of each diluted culture was added to separate wells of a 96-well plate, to which different concentrations of Tax (25–200 μM) had been added, then incubated at 37°C for 24 h. Subsequently, the culture medium was replaced with crystal violet to stain the biofilm for 15 min. The wells were then washed thoroughly with sterile deionized PBS three times. The crystal violet stain in each well was decolorized with 200 μL absolute ethanol and then the OD_595_ value was recorded using a microplate reader.

### Effects of Tax on Mature Biofilms

*S. aureus* biofilms were formed for 24 h at 37°C on the surfaces of the wells of a 96-well plate. Following biofilm formation, different Tax concentrations (25–200 μM) were added to selected wells and the plates were incubated for 24 h at 37°C. The effects of Tax on the mature biofilms were estimated using the crystal violet stain and recorded by a microplate reader.

### FITC-IgG Binding Assay for Staphylococcal Protein A (SpA)

Bacterial cultures grown overnight were diluted 1:100 and cultured in an incubator at 37°C with Tax or DMSO to a concentration of 10^8^ CFU/mL. The Δ*srtA* mutant strain was assayed as a positive control. The bacteria were collected and washed three times in PBS. A 50 μL aliquot of bacterial culture was mixed with an equal volume of FITC-labeled rabbit anti-goat-IgG (1:200, Sigma, United States) then incubated in the dark for 1 h. The bacteria were then washed and suspended in PBS. A multimode microplate reader (Tecan, Spark 20M) was used to measure fluorescence intensity at 535 nm when illuminated at an excitation wavelength of 485 nm.

### Invasion Assay

A549 human lung carcinoma cells were seeded at a density of 2.5 × 10^5^ per well in 24-well plates and placed in an incubator at 37°C in an atmosphere containing 5% CO_2_ for 20 h. Cultures of *S. aureus* were mixed with different concentrations of Tax then cultured at 37°C until an OD_600_ of 1.0 was achieved. The A549 cells were resuspended in medium and aliquots of bacterial suspension were added to each well to a total concentration of 2 × 10^7^°CFU/mL. After incubation for 2 h, the invasion assay was terminated by incubation with 300 μg/mL gentamicin for 30 min. The cells were then lysed after washing and coated on a BHI agar plate. After incubation at 37°C for 12 h, the number of CFUs were counted manually.

### Western Blot Analysis

To evaluate the effect of Tax on SrtA expression, Tax (25–200 μM) was added to different bacterial cultures and incubated overnight at 37°C. Equal quantities of total protein from the bacterial lysates were separated by 12% SDS-PAGE then transferred to a polyvinylidene fluoride (PVDF) membrane using a transblot semidry system. Non-specific binding to the PVDF membrane was prevented by incubating in 5% bovine serum albumin (BSA) overnight at 4°C. The membranes were washed three times with PBST, agitating each time for 5 min, then incubated with rabbit polyclonal antibody (created in the laboratory) against SrtA. Following a further washing in PBST three times, the membranes were incubated with HRP-labeled goat anti-rabbit IgG (Bioworld, China) diluted 1:10,000 in antibody diluent (PBST + 1% BSA) at 37°C for 1 h. Δ*srtA* or *S. aureu*s USA300 without Tax was considered the control group. The cytoplasmic protein ClpP was analyzed as an internal control. Blots were visualized using an enhanced chemiluminescence (ECL) detection system (GE Healthcare, United Kingdom), while bands were quantified using ImageQuant TL software (GE Healthcare).

### Localized Surface Plasmon Resonance

The interaction between Tax and SrtA was determined using an OpenSPR localized surface plasmon resonance (LSPR) instrument (Nicoya, Canada) at 25°C, as described previously (Panneer Selvam et al., 2018; Song et al., 2019). Briefly, SrtA proteins were captured on a COOH chip using a standard amine coupling system. A 200 μL aliquot of blocking buffer was added to rinse the sample ring and to remove any air. After a stable baseline had been achieved, the affinity between Tax and SrtA was measured by injecting 20, 40, 80, 160, or 320 μM Tax. The kinetic parameters of the binding reaction were calculated and visualized using TraceDrawer software (Yang et al., 2011).

### Molecular Docking and Dynamic Simulation

For molecular docking (MD) simulations, Tax (PubChem ID: 439533) was docked onto the SrtA structure of *S. aureus* [Protein Data Bank (PDB) ID: 1T2P] using AutoDock Vina 1.1.2 software, with default parameters (Trott and Olson, 2010). The most appropriate docked pose (conformation) for the Tax-SrtA complex obtained using molecular docking was subject to 25 ns molecular dynamic simulations using Amber14 software (Götz et al., 2012). Preparation of the complex and the molecular dynamic simulation were conducted as previously described (Chan et al., 2017; Niu et al., 2019).

### Murine Model of Pneumonia

To investigate the therapeutic capability of Tax to treat acute pneumonia infection caused by *S. aureus* USA300, 6- to 8-week-old female C57BL/6J mice were infected intranasally with 2 × 10^8^ CFU of *S. aureus* USA300, then held upright for 30 s to ensure each animal inhaled the bacteria into their lungs. Two h post-infection, the mice were subcutaneously injected with Tax (100 mg/kg). Similarly, mice were challenged with Δ*srtA* and sterile PBS containing 0.5% DMSO (control group) as controls. To assess survival, the mice were checked every 12 h for 96 h, and the percentage that had survived was recorded.

For bacterial counts in lung tissue and histopathological examination, mice were infected intranasally with 30 μL (1 × 10^8^ CFUs) of *S. aureus* USA300. Tax (100 mg/kg) was then injected subcutaneously every 12 h after inoculation. The bacterial load in the lungs was determined after the mice in each group were sacrificed 48 h post-infection. The left lung from each animal was removed aseptically and processed to count the bacterial CFUs. The right lung of each mouse was aseptically removed and analyzed by conventional hematoxylin and eosin (H&E) staining using an optical microscope. The ratio of wet to dry lung tissue weight (W/D ratio) was recorded in each case. Wet lung weight was measured 1 min after surface moisture had been removed. Dry lung weight was determined after dehydration at 80°C. The W/D ratio is used to reveal the degree of tissue edema (Xia et al., 2016).

Acute toxicity of Tax was evaluated in mice in accordance with the guidelines for the study of acute toxicity of chemical drugs in China (H-GPT1-1) (Carmichael, 2014). Briefly, each group of 5 female BALB/c mice (6–8 weeks old, weight 18–20 g) was administered a single intraperitoneal injection of 200, 100, or 50 mg per kg bodyweight of Tax, respectively. An injection of PBS represented the control group. Symptoms of poisoning or abnormal behavior were monitored, and survival of the mice in each group was recorded after 72 h.

### Ethics Approval Statement

All animal experiments and surgical procedures were carried out in accordance with guidelines approved by the Animal Welfare and Research Ethics Committee of Jilin University.

### Statistical Analysis

The data were expressed as the mean ± SD for each group in the individual experiments. The experimental data in this study were analyzed using GraphPad Prism 8.0. Statistical significance was accepted as *P*-values < 0.05.

## Results

### Identification of Tax as a Reversible Inhibitor of SrtA

**FIGURE 1:**
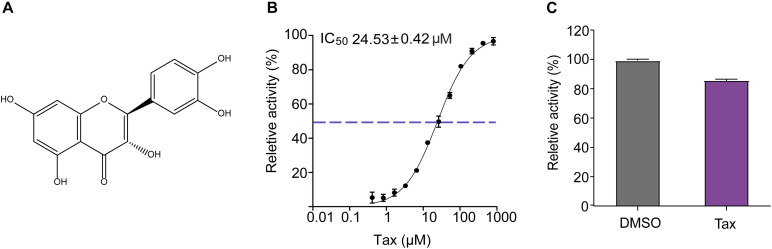
Inhibition of SrtA transpeptidation. **(A)** Chemical structure of Tax. **(B)** Tax inhibits SrtA cleavage of a fluorogenic peptide substrate (Abz-LPATG-Dnp-NH_2_) in a dose-dependent manner *in vitro*. Each reaction condition was assayed in triplicate from which the IC_50_ was determined. **(C)** Recombinant SrtA was treated with 10 × IC_50_ of Tax and then diluted, after which transpeptidation activity was measured using a FRET assay. Untreated SrtA (Control) represented 100% activity.

To identify novel inhibitors of SrtA, an in-house library containing 420 natural compounds was screened, using a FRET assay. The flavonoid Tax was found to display excellent inhibitory properties against *S. aureus* SrtA, with an IC_50_ value of 24.53 ± 0.42 μM (Figures 1A,B). This suggests that Tax is a relatively strong inhibitor of SrtA, compared with previously reported inhibitory small molecules (Oh et al., 2004; Chenna et al., 2010). To further clarify the interacting way of SrtA and Tax, recombinant SrtA was reacted with Tax at a concentration of 10-fold IC_50_. A total of 85.80 ± 0.67% of SrtA activity was subsequently recovered, compared with the control group (DMSO) (Figure 1C). These data demonstrated that Tax was a reversible SrtA inhibitor, and bound non-covalently to the active site of SrtA.

### Tax Has No Significant Inhibitory Effect on the Proliferation of *S. aureus* or Animal Cells

**FIGURE 2:**
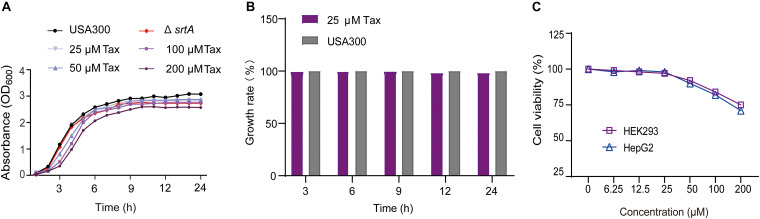
Growth curve and cytotoxicity. **(A)** Growth curves of *S. aureus* USA300 and the Δ*srtA* group with different concentrations of Tax. **(B)** Growth rate of *S. aureus* USA300 and *S. aureus* treated with Tax (25 μM). **(C)** Percent cell viability of HEK293 (purple) and HepG2 (blue) cells were measured using a CCK-8 assay after incubation with Tax for 24 h.

The MIC of Tax against *S. aureus* USA 300 was 512 μg/mL. The results of the growth curve and growth rate showed that Tax had no significant effect on the growth of *S. aureus*, at a concentration of IC_50_ (*P* > 0.05, Figures 2A,B). Safety testing further revealed that Tax displayed no significant cytotoxicity toward HEK293 and HepG2 cells at the concentration (24.53 ± 0.42 μM) required for repression of SrtA (Figure 2C). Taken together, Tax was effective and safe at the concentration required to inhibit SrtA, it was a promising candidate anti-virulence inhibitor and has the potential to be developed into a small molecule drug based on inhibition of SrtA.

### Tax Represses the Adhesion of *S. aureus* to Fibrinogen

**FIGURE 3:**
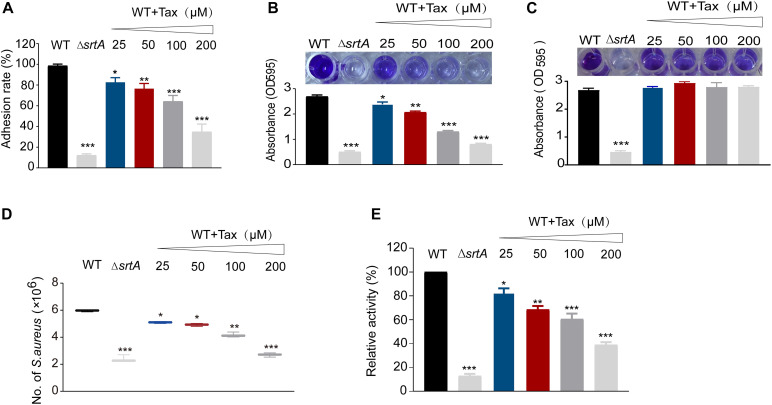
Effect of SrtA inhibitor on virulence-related phenotypes in *S. aureus* Newman strain. **(A)** Impact of Tax on the ability of *S. aureus* to adhere to fibrinogen. **(B)** Biofilm formation of *S. aureus* in the presence of different concentrations of Tax. **(C)** Mature biofilms of *S. aureus* in the presence of different concentrations of Tax. The Δ*srtA* group represented the positive control. **(D)** Tax affects internalization of *S. aureus* into A549 cells. A549 cells were infected with *S. aureus* pretreated with different concentrations of Tax then lysed 2 h post-infection. The number of viable *S. aureus* in the cells was quantified by serial dilution on TSA agar plates. **(E)** Effects of Tax on *S. aureus* protein A (SpA) using FITC-labeled rabbit IgG. Error bars represent means ± SD of three replicates. *P < 0.05, **P < 0.01, ***P < 0.001 calculated with a two-tailed Student’s *t*-test.

To establish whether Tax was able to reduce the adhesion of *S. aureus* to fibrinogen by inhibiting SrtA, a fibrinogen binding assay was performed. As shown in Figure 3A, Tax inhibited the adhesion of *S. aureus* USA300 to fibrinogen in a dose-dependent manner. Compared with the wild-type (WT) control group, 200 μM Tax significantly disrupted the adhesion of bacteria to fibrinogen, with an inhibition rate of 35.09 ± 0.49% (*P* < 0.001). This result reveals that Tax reduced the adhesion of bacteria to fibrinogen via inhibition of SrtA.

### Tax Reduces Biofilm Formation and Fails to Eradicate Mature Biofilm in *S. aureus*

Initial attachment is the first stage of the development of a biofilm. Capture of the multiple surface proteins that are involved is mediated by SrtA (Otto, 2018). Consistent with this, increased expression of SrtA can cause a substantial increase in biofilm biomass in certain staphylococcal strains (Maresso et al., 2007). It can be inferred that Tax can reduce the formation of biofilms. As expected, Tax clearly inhibited biofilm biomass in a concentration-dependent manner. There was an inhibitory effect on biofilm biomass due to the coculture of *S. aureus* with 200 μM Tax, at 29.91 ± 0.51% of the mass of the untreated control. Biofilm formation by the Δ*srtA* group was 18.5 ± 1.02%, indicating that Tax reduces the formation of *S. aureus* biofilms via repression of SrtA activity (Figure 3B). Subsequently, we further evaluated the effects of Tax on mature biofilms, the results showed that different concentrations of Tax (25–200 μM) had no effect on mature biofilms (Figure 3C).

### Tax Suppresses the Invasion of *S. aureus* Into A549 Cells

Since the usual initial point of infection by *S. aureus* is epithelial cells, colonization on the cell surface and their invasion through SrtA-mediated cell surface proteins may result in acute and chronic infection (Gómez et al., 2004). Therefore, the effect of Tax on the internalization of *S. aureus* into A549 cells was investigated. As demonstrated in Figure 3D, the number of bacteria entering cells was reduced significantly following treatment with 200 μM Tax (*P* < 0.001), suggesting that Tax suppresses the internalization of *S. aureus* by inhibition of SrtA, this may be because Tax reduces the anchoring of bacterial surface proteins required for internalization by inhibiting SrtA.

### Tax Influences Anchoring of SpA in *S. aureus*

In *S. aureus*, SrtA can anchor a variety of different surface proteins onto the bacterial envelope (Mazmanian et al., 2002; Falugi et al., 2013). Staphylococcal protein A (SpA) is able to specifically bind the FCγ and Fab regions of IgG. Although there was strong evidence that Tax can inhibit SrtA *in vitro*, it was of great significance to further quantify the surface SpA after treatment with Tax. In the control group, Δ*srtA* displayed weak fluorescence, indicating that it seemed to have completely lost the ability to anchor SpA to the cell wall. After treatment with 200 μM Tax, *S. aureus* displayed a clear decrease in fluorescence, with a relative activity 36.12 ± 1.59% that of the WT group (*p* < 0.001). These results suggests that Tax can reduce the amount of anchored SpA in the cell wall by inhibiting the activity of SrtA (Figure 3E).

### Tax Has No Apparent Influence on SrtA Expression in *S. aureu*s

**FIGURE 4:**
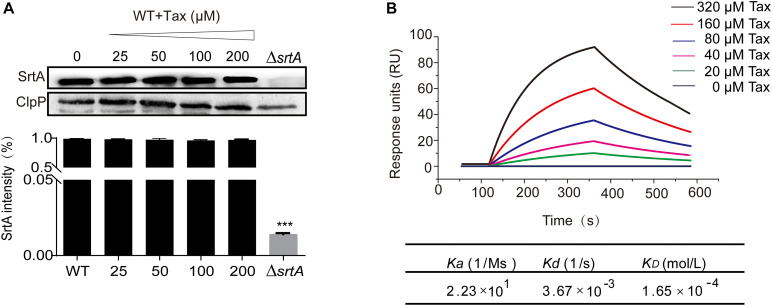
Expression of SrtA in the presence of Tax and the interaction of different concentrations of Tax with SrtA. **(A)** Western blot analysis of SrtA protein from *S. aureus* treated with various concentrations of Tax and grayscale analysis of SrtA protein bands. **(B)** LSPR analysis verified the binding affinity of Tax with SrtA. Error bars represent means ± SD of three replicates. ***P < 0.001 calculated with a two-tailed Student’s *t*-test.

Western blot analysis was used to further evaluate the effect of Tax on SrtA expression Compared with the untreated group, there was no apparent effect on SrtA expression, indicating that Tax effectively blocked the function of SrtA through a mechanism other than inhibition of its expression (Figure 4A).

### Determination of the Molecular Mechanism of the Interaction Between Tax and SrtA

**FIGURE 5:**
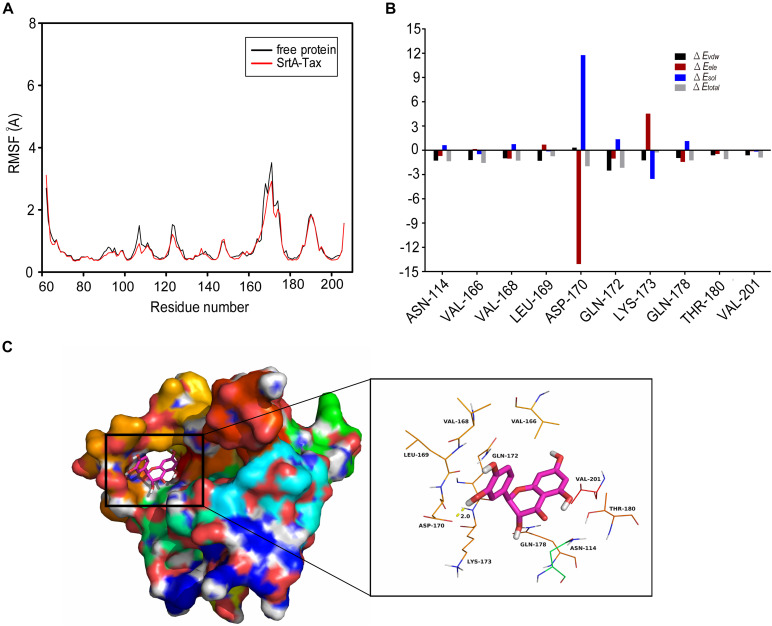
Molecular modeling of the interaction between Tax and SrtA. **(A)** RMSF (Å) graph of free-SrtA (black) and the SrtA-Tax (red) complex during 40-ns MD. **(B)** Docking model of Tax with SrtA during a molecular dynamics simulation. **(C)** Binding free energy decomposition in each residue between Tax and modeled *S. aureus* SrtA.

To explore the interaction between Tax and SrtA, an LSPR experiment was performed. The root mean square fluctuations (RMSF) suggested that SrtA exhibited different flexibility in binding sites with and without Tax (Figure 5A). The results revealed that Tax interacts directly with SrtA in a dose-dependent manner, with a *K*_D_ of 1.65 × 10^–4^ mol/L (Figure 4B). These data indicate that Tax binds directly to SrtA. To investigate the mechanism of the inhibition of SrtA by Tax in more depth, molecular modeling studies were performed. Through analysis of an *in silico* model of the SrtA-Tax complex, it was found that the Asp-170 residue provides a strong electrostatic (Δ*E*_ele_) contribution, with Δ*E*_ele_ < −14.0 kcal/mol (Figure 5B). Asp-170 was found to have the ability to form a strong hydrogen bond with Tax, with a bond length of 2.0 Å (Figure 5B). In addition, Gln-172 provided a significant *van der Waals* force contribution (Δ*E*_vdw_ < −2.5 kcal/mol) (Figure 5B), due to the proximity of Gln-172 to Tax (Figure 5C). Except for Gln-172, the majority of energetic interactions for decomposition stemmed from *van der Waals* interactions, and mostly through the hydrophobic interactions of Val-166, Val-168, Leu-169, and Val-201. Finally, the total binding free energy of the SrtA-Tax complex (Δ*G*_bind_) was calculated, according to the MMGBSA method (Ylilauri and Pentikäinen, 2013), to be −19.2 kcal/mol, indicating that the complexation of Tax with SrtA is strong.

Molecular modeling studies highlight the benefits of performing site-directed mutagenesis studies. Therefore, fluorescence quenching experiments were utilized to determine the binding affinity of Tax with SrtA and SrtA mutants (D170A, Q172A). As displayed in Table 2, the binding constant *K*_A_ for Tax with each SrtA mutant (D170A, Q172A) was significantly lower than that for WT SrtA, indicating that residues Asp-170 and Gln-172 were pivotal binding sites for Tax with SrtA.

**TABLE 2:** The values of the binding constants (*K*_A_) based on fluorescence quenching assay.

**Proteins**	**WT-SrtA**	**D170A**	**Q172A**
*K*_A_ (1 × 10^4^) l/mol	6.4	2.69	3.85
n	0.9682	0.9341	0.8912

Taken together, the results confirm that there is a direct interaction between Tax and SrtA with an inhibitory effect exerted via its direct binding with key residues of SrtA. Confirmation of this interaction allowed additional evaluation of the protective effect of Tax on *S. aureus* infection *in vivo*.

### Tax Protects Mice From MRSA-Induced Pneumonia

Pneumonia caused by MRSA is clinically critical due to its complexity, the high incidence of complications, and high rates of mortality (Michalopoulos and Falagas, 2006). Therefore, the protective effects of Tax were evaluated in a murine model of MRSA-induced pneumonia.

**FIGURE 6:**
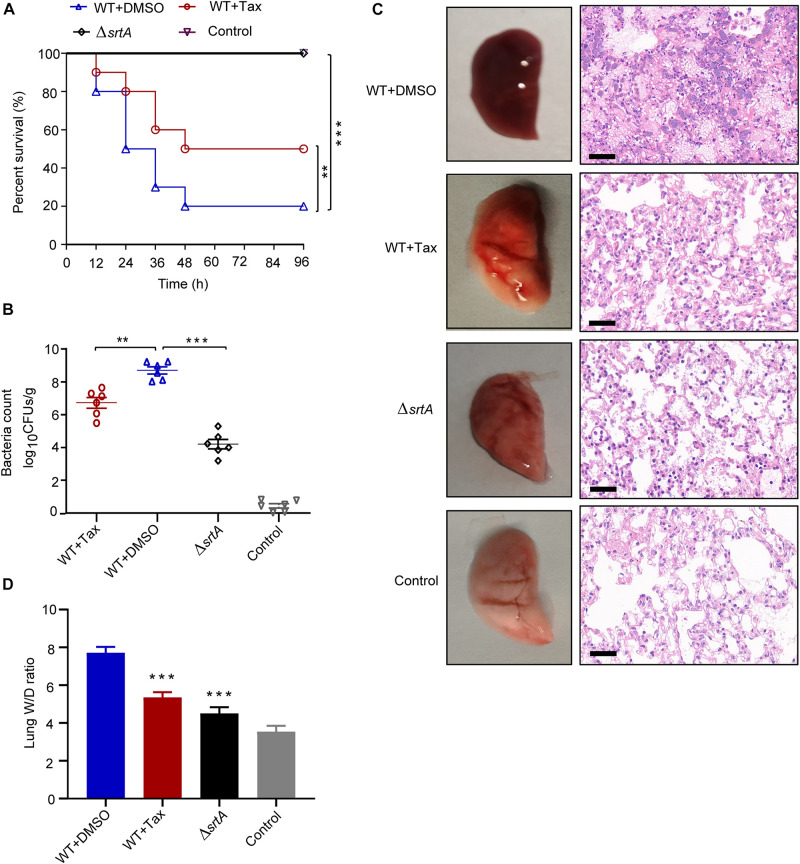
Effect of treatment with Tax on *S. aureus*-induced pneumonia in mice. **(A)** Effect of Tax on the survival of mice (*n* = 10 per group) infected with a lethal dose of *S. aureus*. **(B)** Effect of Tax (100 mg/kg) on bacterial load in the lungs of mice (*n* = 6). **(C)** Gross pathological changes and histopathology of mouse lungs treated with Tax (100 mg/kg/d) or untreated. Scale bar: 50 μm. **(D)** Lung wet-to-dry weight ratio (W/D). Each value represents mean ± SD (*n* = 5 per group). **P < 0.01, ***P < 0.001; Mann-Whitney test, two-tailed. Horizontal bars represent mean values. Animal data were obtained from two separate experiments.

Survival rate was examined by the intranasal administration of lethal doses of *S. aureus* USA300 to mice, with subsequent treatment with Tax every 12 h. The mortality rate was recorded at 12 h intervals for 96 h. As presented in Figure 6A, only 20% of mice survived when challenged with *S. aureus* USA300 96 h post-infection, while infection in the Δ*srtA* group resulted in 100% survival. This indicates that SrtA is key to the pathogenesis of *S. aureus* pneumonia, consistent with previous reports (Cascioferro et al., 2014). Importantly, 100 mg/kg treatment of Tax significantly improved the survival rate of mice to 50% (*p* < 0.01), particularly during the early stages of infection. These findings revealed that Tax provided a strong protective effect against *S. aureus* infection *in vivo.*

The bacterial load in the lungs of the infected group was 8.56 ± 1.34 lg CFU/g (Figure 6B). After treatment with Tax, the bacterial load decreased to 6.73 ± 0.69 lg CFU/g, demonstrating its significant inhibition of *S. aureus* invasion in the lungs.

The pathological changes in mouse lung tissue were further evaluated. Firstly, the appearance of the lung tissue in each group was analyzed. Soft and elastic lung tissue was observed in the control (uninfected) group, whereas lungs infected with *S. aureus* USA300 displayed significant hyperemia and low levels of elasticity, consistent with previous results. Following their treatment with Tax, the lung tissue of infected mice was only slightly red and the extent of congestion was significantly reduced, while elasticity was restored (Figure 6C, left). In addition, histopathological examination demonstrated that the lung tissue of the WT group (*S. aureus*) was clearly hyperemic with a large number of inflammatory cells accumulated within the alveoli. However, treatment with Tax significantly reduced inflammation, as confirmed by the reduced accumulation of inflammatory cells within the alveolar spaces, and relatively complete alveolar structure (Figure 6C, right).

Furthermore, lung W/D ratios, recorded to evaluate the severity of lung injury (Xiao et al., 2008), increased from 3.53 ± 0.16 to 7.71 ± 0.62 after infection with *S. aureus* USA300 (Figure 6D). Conversely, the W/D ratio decreased significantly to 5.35 ± 0.34 in mice treated with Tax (100 mg/kg) (*P* < 0.001).

Taken together, we conclude that Tax attenuates the virulence of *S. aureus in vivo* and provides significant protection against lethal *S. aureus*-induced pneumonia. In addition, the acute toxicity test indicated that no signs of malaise, or tardy or inanimate behavior were observed following intraperitoneal injection of 200 mg/kg Tax.

## Discussion

The evolution, spread, and accumulation of multidrug-resistant (MDR) pathogens, such as MRSA represent a significant hidden risk to human public health (Skaar, 2010). Recent studies have shown that the proportion of MRSA isolated from patients is increasing, leading to significant incidence rate and mortality (Iwata et al., 2020). Antibiotics identified from other microorganisms have always been the primary weapon to combat bacterial infection (Rasko and Sperandio, 2010). These antibiotics rely on disrupting the key components in bacterial synthesis and assembly such as cell wall synthesis, DNA replication and protein synthesis (Mann, 2003). The effective bactericidal effect of these antibiotics also caused great survival pressure on the pathogens, making the rapid production of drug-resistant bacteria (Werner et al., 2008; Rasko and Sperandio, 2010). Therefore, it is imperative to develop alternatives to antibiotics which can not only inhibit infection but also avoid the production of drug-resistant bacteria. A promising strategy is to develop antivirulence therapies. The purpose of antivirulence strategies is not to kill pathogens directly, but to disarm them and prevent their attack on the host. This would not, therefore, exert selective pressure and so reduces the risk of drug resistance to a great extent (Hou et al., 2018). Moreover, a significant advantage of antivirulence strategies is that they reduce the damage to host microbiota, overcoming the adverse reactions and serious side effects of antibiotic therapies (Buroni and Chiarelli, 2020).

SrtA can recognize, cleave, and anchor specific LPXTG-containing proteins which contribute to the bacterial infection process (Si et al., 2016), many of those surface proteins mediate bacterial colonization to host tissues and cells, the formation of biofilms and evasion of the immune response, etc. (Geoghegan and Foster, 2017). In addition, SrtA is constitutively expressed in all clinical isolates (Marraffini et al., 2006; Foster et al., 2014). Therefore, SrtA has long been recognized to be an important virulence factor of *S. aureus* and a promising pharmacological target of antivirulence strategies.

**FIGURE 7:**
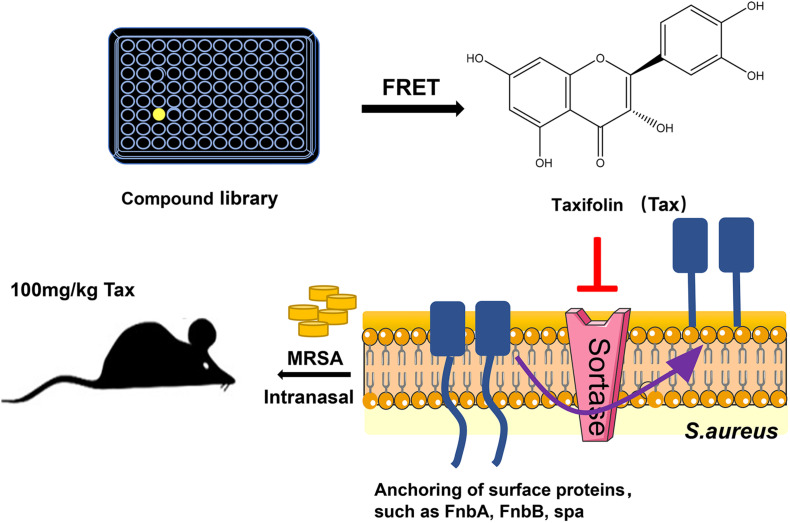
Experimental flow chart.

The experimental flow chart is shown in Figure 7. We identified the natural compound Tax that could block SrtA activity, with an IC_50_ of 24.53 ± 0.42 μM. Inhibitors of SrtA can be categorized as either covalent or non-covalent (Jackson et al., 2017; Jaudzems et al., 2020). The formation of covalent bonds occurs via the reaction of an inhibitor with the cysteine active site (Hou et al., 2018; Barthels et al., 2020). We found that Tax is a non-covalent inhibitor that binds reversibly to SrtA. Reversible inhibitors might be a promising alternative, which overcome the drawbacks of the irreversible inhibitors, such as high toxicity, non-recoverability, non-repairability and other side effects (Beck et al., 2012). Importantly, Tax exhibited no cytotoxicity to mammalian cells (HEK293 and HepG2) at the concentration required to inhibit SrtA. This satisfies the main criteria for an ideal antibacterial agent: low selectivity pressure that avoids the generation of drug resistance (Schneewind and Missiakas, 2019). *S. aureus* is considered notorious due to its extraordinary capability to create a biofilm (Oliveira et al., 2018), a surface-attached encasement of cells and matrix. Biofilms lead to increased antibiotic resistance and evasion of the host immune system, increasing the complexity of the existing problem of antibiotic resistance (Vermote and Van Calenbergh, 2017; Graf et al., 2019). In the present study, our results showed that the co-incubation of *S. aureus* with Tax led to decreased biofilm formation but did not affect the mature biofilm, which indicates that SrtA inhibited the formation of biofilm by inhibiting the anchoring of surface protein required in the adhesion stage of biofilm (Ming et al., 2017; Vazquez-Armenta et al., 2018). Moreover, the MD simulation and mutagenesis study indicated that Tax binds to the binding pocket of SrtA, depending mostly on hydrogen bonding, and electrostatic and *van der Waals* interactions, in which Asp-170 and Gln-172 play an important role.

Bacterial strains with a mutation of SrtA have exhibited attenuated virulence in a mouse infection model (Chen et al., 2005), suggesting that SrtA performs a critical role in staphylococcal pneumonia. Therefore, inhibition of surface protein anchoring through the interference of SrtA should affect the ability of *S. aureus* to establish pulmonary infection. As expected, we observed that Tax significantly protected mice from challenge by lethal doses of *S. aureus* USA300, resulting in a reduction in bacterial burden in lung tissue. In addition, the safety of Tax was verified by acute toxicity testing which indicated that Tax has the potential for further development and clinical application.

Collectively, the present study reports on the discovery that the natural compound Tax is a specific SrtA inhibitor that is safe and efficacious, able to attenuate MRSA virulence *in vitro* and *in vivo* and the potential for development into a novel antivirulence agent. In addition, sortases possess an LPXTG sorting signal which is present in almost all strains of low G + C Gram-positive bacteria (Sillanpää et al., 2010; Spirig et al., 2011; van Harten et al., 2017; Kang et al., 2020). This suggests that SrtA inhibitors have the potential for further development and could be widely used in the treatment of multiple Gram-positive infections.

## Data Availability Statement

The original contributions presented in the study are included in the article/supplementary material, further inquiries can be directed to the corresponding author/s.

## Ethics Statement

The animal study was reviewed and approved by Institutional Animal Care and Use Committee (IACUC) of Jilin University.

## Author Contributions

QL, GW, and DW conceived and designed the experiments. LW, SJ, and SG performed and analyzed the experiments. LS, HQ, and KW performed the molecular dynamics simulation. LW prepared the original manuscript and revised the manuscript.

## Conflict of Interest

The authors declare that the research was conducted in the absence of any commercial or financial relationships that could be construed as a potential conflict of interest.
